# Simple Physical Mixing of Graphitic Wood-Derived Carbon For High-Performance Ni(OH)_2_ Electrodes: A Sustainable Strategy Beyond Metal Additives

**DOI:** 10.1021/acsomega.5c11266

**Published:** 2025-12-22

**Authors:** Xingyan Zhang, Sadaf Saeedi Garakani, Gunder Karlsson, Dag Noréus

**Affiliations:** Department of Chemistry, 7675 Stockholm University, SE 106 91 Stockholm, Sweden

## Abstract

The replacement of expensive metal powders in Ni­(OH)_2_-based cathodes is essential for reducing cost and environmental impact in aqueous Ni–Zn batteries. This work investigates graphitic wood-derived carbon (GWC) as a sustainable conductive additive to boost the performance of Ni­(OH)_2_ pasted electrodes prepared by a simple physical mixing process. A number of graphitic wood-derived carbon qualities are explored as functional additives replacing expensive cobalt and nickel powder additives while maintaining the electrochemical performance of Ni­(OH)_2_ electrodes in aqueous rechargeable Ni–Zn batteries. The GWC offers high electrical conductivity and a unique microsized particulate morphology. Optimizing the GWC content to 25 wt % yields a specific capacity of 284.2 mAh g^–1^ at 0.2C, which is better than that of electrodes containing only Ni­(OH)_2_, with Ni/Co powders, or commercial carbon black. Furthermore, the open-circuit voltage hysteresis and state of charge are studied to understand the charge/discharge process, suggesting that GWC is an effective alternative to expensive metal powders, providing a low-cost and sustainable strategy for improving Ni­(OH)_2_-based electrodes through a straightforward manufacturing process.

## Graphical abstract

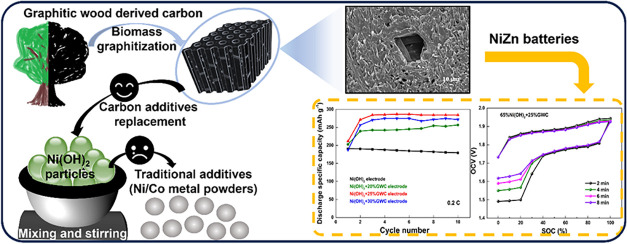

## 1 Introduction

Among Ni-based alkaline batteries, the Ni–Zn battery stands out as a promising choice, considering performance, cost, safety, and sustainability, which has been used for a long time and will continue to be revitalized.^1–3^ However, despite its possible advantages such as high energy and power density, high depth of discharge, low self-discharge, long cycle life, etc., the amount of nickel and cobalt metal powder used in the electrode preparation process ought to be reduced as it leads to high costs and environmental burdens.^4,5^ For the Ni-based electrode of the Ni–Zn battery, nickel hydroxide (Ni­(OH)_2_) is in practice the β-variety. There is also a metastable allotrope, alfa nickel hydroxide, which is not used today but efforts are done trying to realize it as it offers a substantially increased capacity.^6,7^ The reversible reactions occurring involve two redox pairs: α-Ni­(OH)_2_/γ-NiOOH and β-Ni­(OH)_2_/β-NiOOH. Among them, the phase transition from α-Ni­(OH)_2_ to γ-NiOOH provides higher theoretical specific capacitance due to the higher Ni oxidation state approaching Ni^4+^ in γ-NiOOH.^8,9^ However, α-Ni­(OH)_2_ is unstable in aqueous electrolytes and easily transforms into β-phase. To stabilize the α-Ni­(OH)_2_ crystal phase, dopants and electrolyte additives have been tried.^10–13^ The poor electronic conductivity of both the α/γ and the β/β redox couples are amended with a metallic current collector usually in the form of nickel foam or nickel-plated expanded metal strips that allows for short distances from the Ni-hydroxide particles to the current collector. Fine powdered INCO Ni-powder is often also added to improve conductivity. Cobalt addition to create a conducting CoOOH network when the cells are formed is also used.^14–17^ Commercial Ni­(OH)_2_ electrodes rely heavily on cobalt additives, which are not only expensive but also have supply chain and ethical issues. The roles of cobalt are to form a conductive CoOOH network, inhibit the oxygen evolution reaction (OER) at the end of charging, stabilize the β/β reaction, and slow down the expansion and pulverization of the electrode. Without cobalt, the Ni­(OH)_2_ electrode will suffer severe volume changes and mechanical stress during cycling, especially at high depths of discharge and in the presence of α/γ phase transition, leading to the shedding of active materials and capacity decay. This is a key mechanical engineering problem. Slow but continuous progress has been made in developing materials, and how they can be implemented in actual industrial production.^18–20^ Increasing the practical capacity in Ni–Zn batteries concomitant with reducing the content of added nickel and cobalt will help us to ensure more sustainable energy solutions. This topic has become more attractive in Europe, as reflected by the present EU Horizon 2020 LOLABAT project involving 17 partners in 7 countries. At present, pure or simply modified Ni­(OH)_2_ is mainly used in industrial production, and an appropriate amount of metallic nickel or cobalt powder is added to increase its conductivity and stability.^21^ Replacing these metal powders with a sustainable material that addresses both conductivity and mechanical stability remains a key challenge. From an industrial perspective simple directly physical mixing Ni­(OH)_2_ and additives will effectively lower production costs.^22–24^

Generally, carbon-based materials are effective candidates to improve conductivity and reduce cost.^25–27^ A few recent reports describes the enhancement effect of carbon additives through physical mixing processes.^28–31^ Maiyalagan et.al studied the effect of four kinds of carbon materials (multiwalled carbon nanotube, graphene, acetylene black, and Vulcan carbon) on the Fe_2_O_3_ electrodes to limit the hydrogen evolution reaction for rechargeable alkaline Fe-air batteries, thus troubleshooting the existing capacity fading problems and scalability.^32^ Wheeler’s group reported that different carbon additives with different Scott densities and specific surface areas can affect the electrolytic manganese dioxide electrodes for alkaline Mn–Zn batteries.^33^ Up to now, some researchers have reported the synergistic effect of various carbon-based materials with Ni­(OH)_2_ paste electrodes. Chang et.al synthesized CoO/RGO (Reduced Graphene Oxide) as an additive to nickel hydroxide electrodes, which not only reduces the amount of CoO added but also has high rate capability, indicating that the addition of RGO can significantly enhance reversibility, proton diffusion, and conductivity.^34^ Lota’s group even reported five kinds of carbon materials (flake graphite, different multiwalled carbon nanotubes) on the electrochemical performance of pasted nickel electrodes to explore the influence.^35^ The morphology and size of the carbon additive are critical as they determine the quality of the conductive network formed within the composite electrode. Biomass carbon has received a lot of attention in recent years due to its abundant, renewable sources and unique structure.^36–38^ Among numerous biocarbon materials, wood-derived carbon that can achieve high graphitization degrees and conductivity after high-temperature treatment is a very promising candidate for various energy storage devices.^39–41^ However, its potential as a multifunctional additive to solve both conductivity and mechanical issues in industrial electrodes is unexplored.

The main purpose of this work is to reduce the amount of metal powders in the Ni­(OH)_2_ electrode of commercial Ni–Zn batteries and to further increase the cycling stability by using graphitic wood-derived carbon (GWC) through a physical mixing process. This three-dimensional interconnected GWC not only provides excellent electronic conductivity (replacing the conductive function of metal powder), but its unique natural structure may also provide mechanical support for the electrode and help mitigate volume expansion (replacing the stabilizing function of cobalt), which may provide an industrial reference for achieving low-cobalt or cobalt-free and significantly improving the cycle life of the electrode. We explore the effects of different carbon materials on the performance of the nickel electrode and optimize the best candidates. Owing to the advantages of the Ni­(OH)_2_ and GWC, the obtained electrode has a remarkable electrochemical performance. Furthermore, the open-circuit voltage hysteresis and state of charge are studied to understand the ion adsorption and intercalation reactions during the charge/discharge process. This work offers an effective and sustainable strategy to improve the electrochemical performance of low-cobalt commercial Ni­(OH)_2_ electrodes by adding an appropriate amount of graphitic wood-derived carbon to replace the industrially expensive metal powder, which provides a simple and industrially feasible battery electrode processing solution.

## 2 Experimental Section

### 2.1 Preparation of Graphitic Wood-Derived Carbon (GWC)

First, Balsa wood (purchased from Material A B, Sweden) was cut into thin slices with controllable thickness by cutting equipment. All wood membranes were dried at 80 °C in an oven overnight before chemical extraction. For removing hemicellulose and lignin, an acetate buffer solution (pH 4.6) with NaCl was applied at 80 °C for 6 h. Then, the samples were washed by using deionized water and dried at room temperature under a vacuum beneath a glass slide. For carbonization, the wood membrane was heated to 300 °C for 1 h; then, it was heated to 600 °C for another 1 h; finally, it was heated to 900 °C and was retained for again 1 h. Afterward, the furnace was cooled down to ambient temperature in 6 h. During the whole process, the furnace vacuum was kept constant at 13 mbar in the N_2_ atmosphere. All wood membranes were graphitized under vacuum using a national spark plasma sintering (SPS, Dr Sinter 825, Fuji Electronics, Japan). For the SPS process, a rapid heating rate of up to 100 °C min-1 was performed and a final temperature of 2000 °C with a holding time of 10 min was realized.

### 2.2 Preparation of Composite Electrode

To obtain the composite electrode, the Ni­(OH)_2_ (purchased from Henan Kelong New Energy Co., Ltd.), graphitic wood-derived carbon, and polyvinylidene fluoride (PVDF) in a weight ratio of (90-*x*): *x*: 10 were directly mixed by stirring and then dispersed in *N*-Methyl pyrrolidone (NMP) to form a paste. Here, the *x* is the amount of graphitic wood-derived carbon, and it is 20, 25, and 30, respectively. Finally, the mixture was coated on a piece of nickel felt (∼1 × 3 cm^2^) and dried at 120 °C under vacuum overnight.

For comparison, some cells are assembled with different carbons. The Ni­(OH)_2_, carbon additive, and PVDF in a weight ratio of 7:2:1 were mixed and dispersed in NMP followed by drying at 120 °C under vacuum overnight. The S, N-codoped wood carbon was prepared without SPS treatment.^42^ The Graphitic carbon black 2 was treated with the same conditions as GWC by using SPS, and this carbon black 2 is another carbon black from a company (Stack of Fire AB, Sweden). Graphene-1 and Graphene-2 were obtained from 2D fab and Graphmatech companies in Sweden, respectively.

### 2.3 Characterization

The morphologies of the materials were observed by scanning electron microscopy (SEM, JSM-7000F, Japan), field emission scanning electron microscopy (FESEM, Zeiss Ultra 55 GEMINI, Germany), and transmission electron microscope (TEM, JEM-2100F, Japan). The crystal structures were investigated by an X-ray diffractometer (XRD, D8 DISCOVER, Bruker) using Cu Kα radiation. The carbon structure was characterized by Raman spectra using a LabRAM HR800 spectrometer (HORIBA, France) with an Nd: YAG laser at an excitation wavelength of 532 nm and a power of 50 mW. The surface and pore characterization of the wood-derived carbon were characterized by N_2_ adsorption/desorption measurements using an ASAP 2020 surface area and porosity analyzer (Micromeritics).

### 2.4 Electrochemical Measurements

For the electrode, the cyclic voltammetry (CV) curves were tested in a three-electrode system by employing the Pt wire and Hg/HgO as the counter and reference electrodes in a 6 M KOH solution, respectively.

The Ni–Zn cell was assembled by using the Ni­(OH)_2_/carbon additive as the cathode and the Zn plate as the anode. Before testing, the cell was immersed in the 6 M KOH electrolyte for 24 h. All the cells were tested at 0.2C with a 20% overcharge for several cycles for activation. The electrochemical performances were tested by using a Battery Testing System (Land, China). The capacities of all the cells were calculated based on the mass of Ni­(OH)_2_. All open-circuit voltage hysteresis data were obtained by waiting for different time after a charging interval of 10%. The electrochemical impedance spectroscopy (EIS) and the open-circuit voltage were tested to analyze the potential of the prepared electrodes by using a VSP300 electrochemical workstation (Biologic, France).

## 3 Results and Discussion

The scanning electron microscopy (SEM) images of the obtained graphitic wood-derived carbon are shown in Figure 1a–d. After carbonization and graphitization, the GWC sample retains the natural original skeleton morphology of the raw wood, presenting a porous channel structure on a micrometer scale, which may help form an efficient conductive network. The transmission electron microscope (TEM) images of the GWC sample at different magnifications are presented in Figure 1e,f. It can be clearly seen that amorphous structures coexist with partially ordered structures. The high-resolution TEM image of the ordered lattice structure shown in Figure 1f reveals the existence of a high degree of graphitic structure. X-ray diffraction (XRD) measurement (Figure 1g) further reveals the structural characterization of the GWC sample. The GWC sample shows a well-developed graphitic stacking peak at 2θ of ∼26.5° and a broad weak peak at 2θ∼42° due to the formation of a high degree of interlayer channel condensation, indicating higher electrical conductivity. The electrical conductivity of the GWC sample was measured using a four-probe setup and was found to be 1.1 × 10^5^ mS cm^–1^ (Table S1, Supporting Information, (SI†)), which is thousands of times higher than that of commercial activated carbon (20∼100 mS cm^–1^ for TF-B520).^43^ Compared with the conductivities of some biocarbons reported in the literature (0.62–38 mS cm^–1^), the conductivity of the GWC sample is also clearly higher making it comparable to metal powder, and thus it could contribute to achieving great rate capability. Nonetheless, compared to pristine, carbonized, and low-temperature graphitized wood-derived carbon studied in the previous work, this GWC shows the highest electrical conductivity and dense structure after sacrificing the high specific surface area resulting from the porous structure.^44,45^

**Figure 1:**
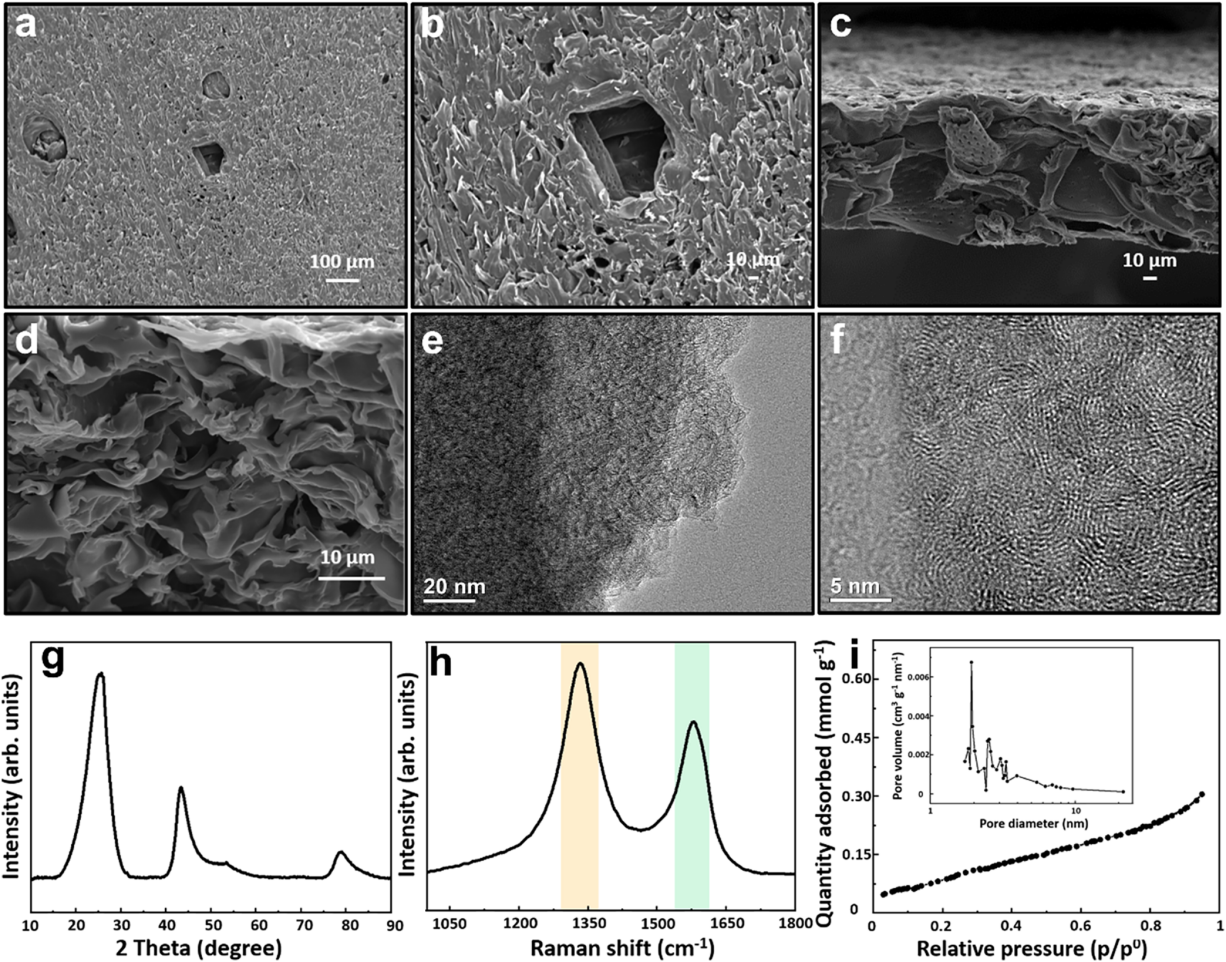
Characterizations of GWC: (a and b) SEM images of top-view, (c and d) SEM images of cross-view, (e and f) TEM images, (g) XRD pattern, (h) Raman spectrum, and (i) N_2_ sorption isotherm, the inset is the pore size distribution curve.

Raman spectrum of the GWC sample indicates the microstructure of the produced materials (Figure 1h). There are two peaks containing a G-band located around 1590 cm^–1^ corresponding to the graphite structure and a D-band located around 1340 cm^–1^ depending on the disorder and defects in the carbon structures. The high ratio of the intensities of the two peaks (*I*_D_/*I*_G_ = 1.13) indicates large defects existing in the GWC as well as a high content of amorphous carbon.^26^ Furthermore, the nitrogen sorption measurement was carried out to investigate the specific surface area and pore structural properties of the GWC sample (Figure 1i). This curve suggests the presence of mesopores and even macropores in the GWC sample. In accordance with the N_2_ adsorption amounts, the GWC sample exhibits a specific surface area of around 8 m^2^ g^–1^, and the total pore volume is 0.01023 cm^3^ g^–1^. As shown in the inset in Figure 1i, the GWC sample exhibits a weak hierarchical porous structure with a pore size distribution mainly in the range of 1∼10 nm, which is consistent with the dense structure observed by SEM. This compact structure provides high electrical conductivity and corrosion resistance, which is ideal for additives. In addition, the still-existing natural pore channels of the wood could provide fast ionic transport paths, allowing for good electrochemical performance.

For the Ni­(OH)_2_ powders, the SEM images shown in Figure 2a–c present their morphology structure conducting of 1–10 μm particles. The surfaces of these particles consist of staggered stacked sheets, forming a three-dimensional overlapping framework that facilitates electron transfer and ion diffusion. The crystal structure was also confirmed by XRD patterns. In Figure 2d, the strong peaks at 19.1, 38.45, 51.95, 59.15, 62.75, 69.5, and 72.8° correspond to (0 0 1), (1 0 1), (1 0 2), (1 1 0), (1 1 1), (2 0 0), and (2 0 1) planes of the hexagonal β-Ni­(OH)_2_ powders (JCPDS card No. 14-0117). When the material is prepared into a pasted electrode, the main crystalline phase does not change, except for the addition of several signals corresponding to the current collector. The peaks at 44.6, 51.9, and 76.4° correspond to (1 1 1), (2 0 0), and (2 2 0) planes of the nickel felt substrate (marked pink, JCPDS card No. 04-0850), and the weak peaks at 38.4, 44.7, 65 and 78.1° correspond to (1 1 1), (2 0 0), (2 2 0), and (3 1 1) planes of the aluminum holder (marked blue, JCPDS card No. 65-2869) that is employed to fix the sample during the XRD measurement.

**Figure 2:**
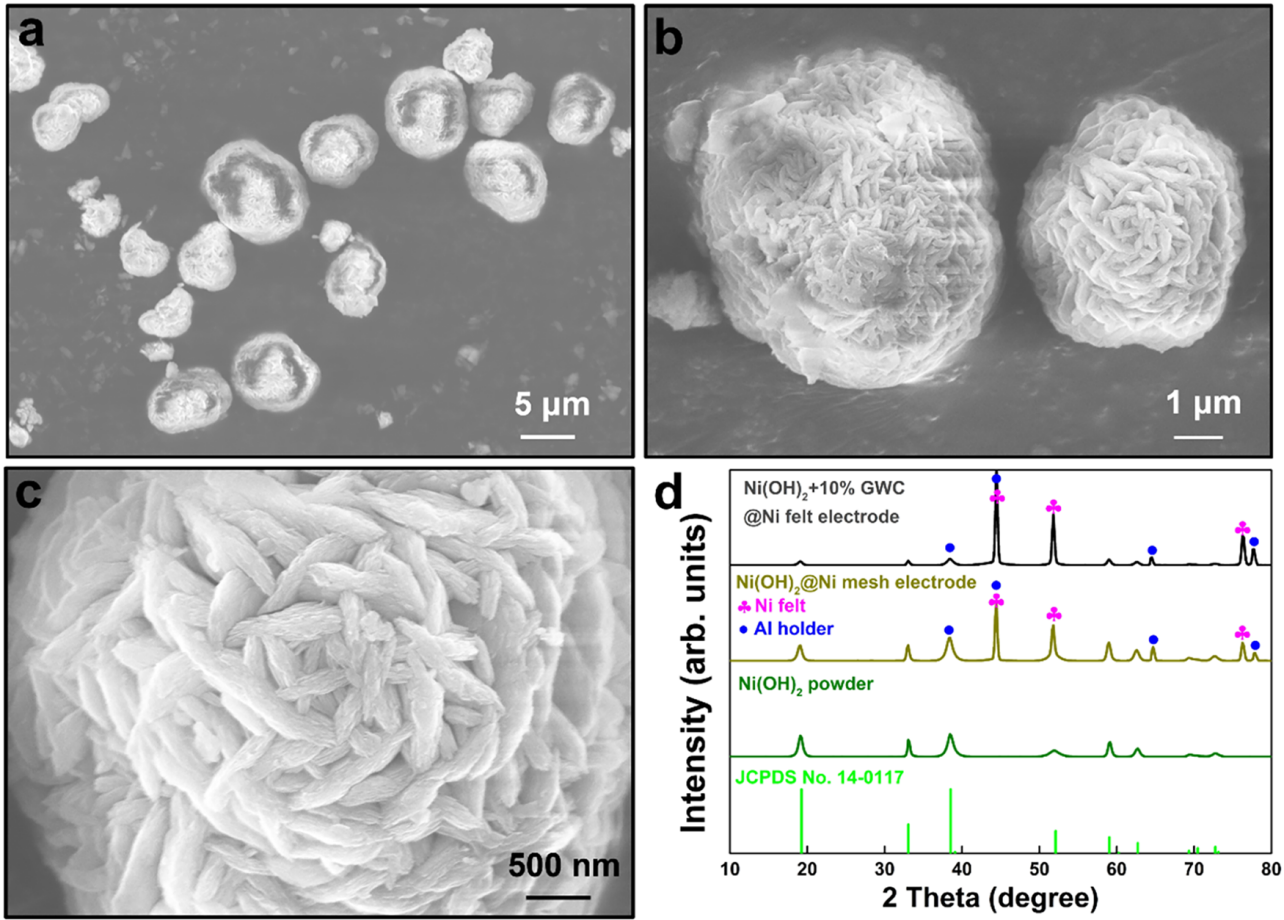
(a-c) SEM images of Ni­(OH)_2_ powders, and (d) XRD patterns of the materials and pasted electrodes.

To explore the practical potential of the GWC/Ni­(OH)_2_ electrode, an aqueous NiZn cell was assembled by using the GWC/Ni­(OH)_2_ electrode and a Zn plate. Figure 3a shows the typical charge and discharge curves of different samples with/without GWC additives at 0.2C for 10 cycles. As shown in Figure 3a, the Ni­(OH)_2_+20%GWC//Zn cell presents a lower end-of-charge voltage and better cycling stability, which demonstrates that the GWC additives decrease the oxygen overvoltage. As shown in Figure 3b, wide plateaus of about 1.6–1.8 V are observed in the two discharge curves at 0.2C of the 10th cycle, corresponding to the redox reaction of Ni^3+^/Ni^2+^. It can be noted that the Ni­(OH)_2_+20%GWC//Zn cell has a higher discharge voltage, which also indicates higher conductivity. After calculations (based on the effective mass of Ni­(OH)_2_ in the cathode), it was observed that the Ni­(OH)_2_+20%GWC//Zn cell can reach a maximum capacity of 256.6 mAh g^–1^ at 0.2C whereas that of the Ni­(OH)_2_//Zn cell is 179.4 mAh g^–1^. Electrochemical impedance spectra (EIS) measurement was carried out to explore the resistance of the electrode. Figure 3c shows the compared Nyquist plots of the two cells. After adding the GWC, the cell has obviously lower charge transfer resistance (semicircle in the high-frequency region) and diffusion resistance (the straight line in the low-frequency region), reflecting the enhanced electron conductivity, which also demonstrates the relatively easier electrochemical reaction. Figure 3d shows the discharge capacity and efficiency of the two cells at different current densities from 0.2 to 10 C for several cycles to study the cycling stability. Accordingly, the cycling stability of the electrode with 20% GWCs is much improved. Also, Figure S1 (SI†) shows the compared performances of two cells with Ni­(OH)_2_ and Ni­(OH)_2_+25%GWC electrodes, respectively. Clearly, the cell with the GWC has a higher capacity and better cycling stability, as mentioned above, revealing the enhancements after the addition of GWC.

**Figure 3:**
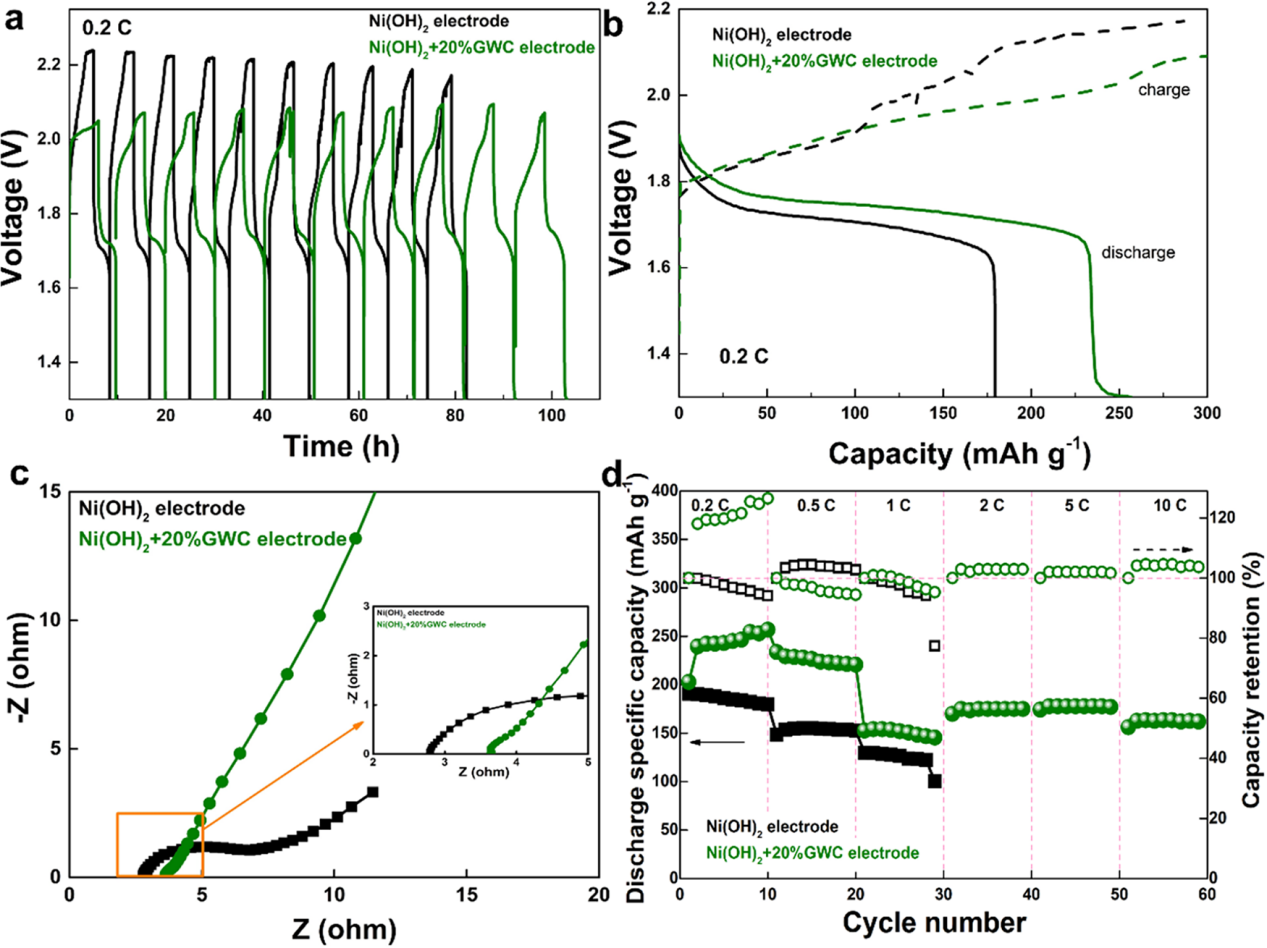
Compared electrochemical performance of electrodes with/without GWC additives: (a) the cycling curves, (b) the compared charge/discharge curves of the 10th cycle, (c) compared Nyquist plots, and the inset is a partial enlargement, and (d) the cycling stability at different current densities.

The content of GWC can influence the performance of the cell. Figure 4 shows the performance of Ni­(OH)_2_ with different GWC contents for pasted electrodes. In Figure 4a, it can be noted that, as the GWC content increases, the capacity of the corresponding cell first increases and then decreases. When the GWC content is 25 wt %, the cell has the largest capacity, reaching up to 284.2 mAh g^–1^. Obviously, the GWC addition can improve the conductivity of the overall electrode and the material utilization of Ni­(OH)_2_. Furthermore, for the Ni­(OH)_2_+25%GWC electrode, the cutoff charge voltage is lower and the discharge plateau is higher than other electrodes, indicating that it has better chargeability and lower intrinsic resistance. Compared with the results in Figure 3b, it can be further confirmed that the GWC addition improves the conductivity and capacity of the overall electrode. However, this improvement is limited. When the GWC content increases to 30 wt %, the discharge capacity decreases because the mass of effective active material is diluted. In other words, the ratio of active material, binder, and conductive carbon has a subtle effect on electrode performance. However, enhanced conductivity additives can optimize the conductive network, thereby improving the utilization of active materials and reducing the capacity reduction caused by mass dilution. A suitable ratio improves both electronic and ionic conductivities between the electrode.^46^ As shown in Figure 4b, the discharge capacities of these cells show a consistent trend, and the Ni­(OH)_2_+25%GWC//Zn cell has the best electrochemical performance. At the same time, although the cycle number is limited, it still shows the best stability, which indicates that the appropriate content of the additive can further boost the practical properties. Also, the relatively long cycling stability at 1 C was detected. As shown in Figure S2 (SI†), the significant capacity decay began to appear after ten cycles of the charge/discharge process, which may be due to factors including the possible precipitation of zinc species on the surface of the Zn counter electrode after long-term testing (because all tests were conducted continuously, after long-term testing, a large amount of salt accumulates on the surface of the Zn counter electrode, as shown in Figure S3, SI†), which could inhibit the further reaction. the large amount of precipitation of zinc dendrites Therefore, to improve the overall conductivity of the electrode and the utilization rate of active materials, the amount of GWC as a conductive addition needs to be adjusted, which is controlled to be 25 wt % in our observation.

**Figure 4:**
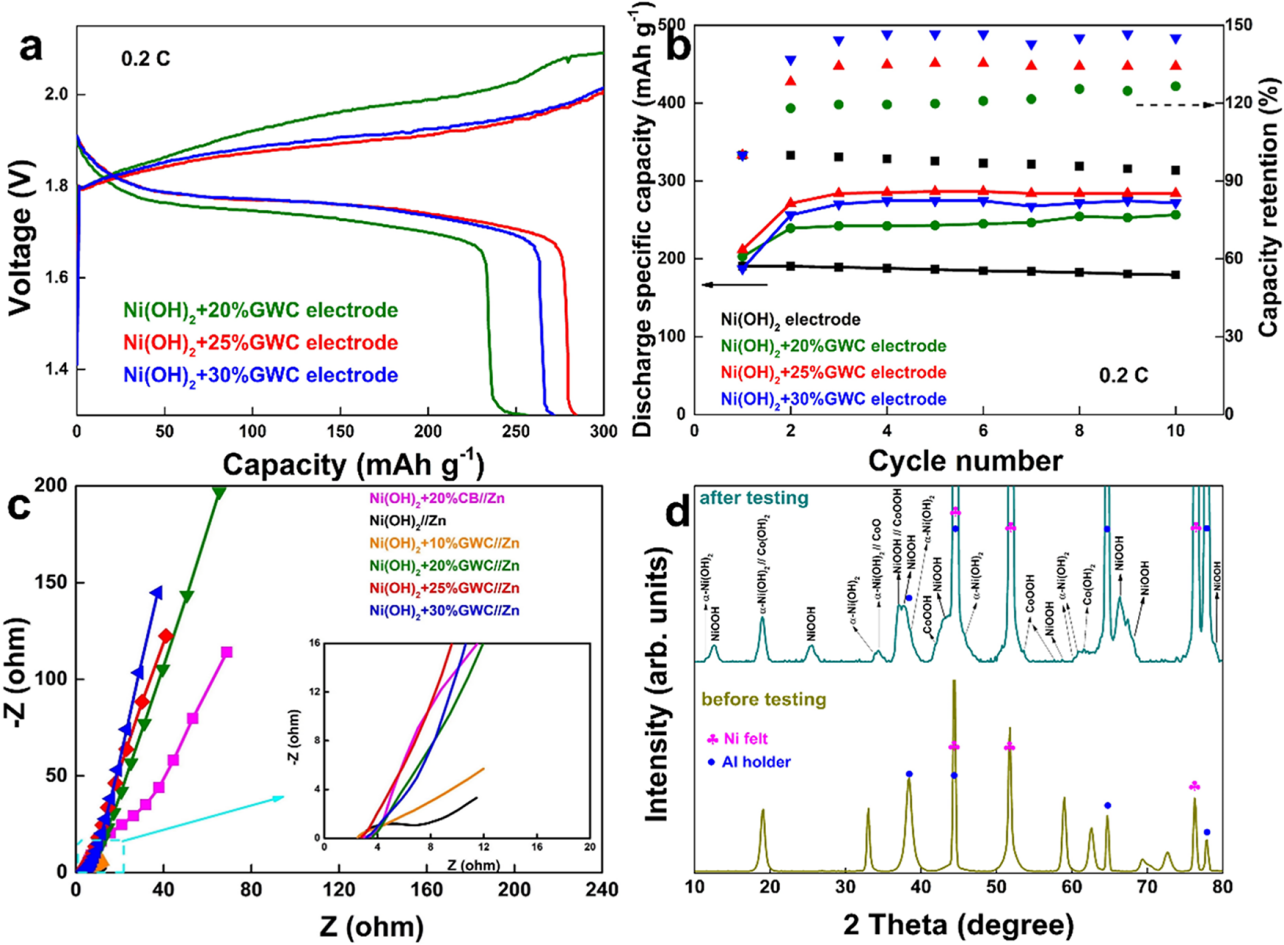
(a) charge/discharge curves of different electrodes with different carbon contents at 0.2C, (b) discharge specific capacities of electrodes with/without different contents of GWC at 0.2C for 10 cycles, (c) Nyquist plots of different cells with different carbon, and (d) XRD patterns of the Ni­(OH)_2_ electrode without carbon addition before and after electrochemical testing.

EIS measurements further confirm the electron kinetic properties (Figure 4c). With the increase in the carbon content, the charge transfer resistance decreases significantly. But when the carbon content increases to a certain value, the increase begins to become smaller and then there is almost no change. In addition, the slope of the straight line represents the diffusion of charges in the electrode material. The larger the value, the higher the diffusion rate. It can be seen clearly that the slope has increased, tending to be perpendicular to the *x*-axis, showing the capacitive characteristics and ultrahigh conductivity of carbon materials as the carbon content increases. Thus, we can further confirm that the GWC addition can improve the conductivity of the overall electrode to boost the properties of the cell. Besides, we also compared the Nyquist plots of NiZn cells with the different Ni-electrodes containing the same carbon content but different carbon sources (GWC and commercial carbon black (CB)). According to the comparison, the Ni­(OH)_2_+20%GWC//Zn cell has obviously lower charge transfer resistance and higher diffusion rate than that of the Ni­(OH)_2_+20%CB//Zn cell, indicating better performance of GWC than that of commercial CB. Generally, when the activated material is diluted with carbon, the distance that electrolyte ions OH^–^ move will be longer. However, GWC has higher conductivity and faster ion transfer rate, especially compared with CB which takes up more space than GWC, so after adding carbon materials, the Ni­(OH)_2_+20%GWC electrode exhibits better electrochemical behavior than the Ni­(OH)_2_+20%CB electrode and the original Ni­(OH)_2_ electrode. Furthermore, as shown in Figure S4 (SI†), the electrochemical performances of cells with different commercial CB contents were studied. Obviously, compared to the electrode without added carbon material (as shown in Figure 3), the electrode containing commercial CB also showed higher discharge-specific capacity and better cycling stability like those of the samples containing GWC. In addition, in Figure S5 (SI†), electrodes containing nickel and cobalt metal powders were also studied. According to the test results compared with electrodes containing only nickel hydroxide, their capacity was not significantly improved but their stability was significantly better. The above results all show that adding GWC can significantly improve the conductivity (replacing the conductive function of metal powders), capacity and stability (replacing the stabilizing function of cobalt) of Ni­(OH)_2_ electrodes to achieving low-cobalt even cobalt-free production, further indicating that it is an excellent possibility to replace metal powder in the industry.

As shown in Figure S6 (SI†), XRD patterns of the Ni­(OH)_2_+10%GWC electrode are presented. It is difficult to see the phase peak position changes of the Ni­(OH)_2_+10%GWC electrode after the electrochemical testing due to the strong signal from the nickel substrate and the interference from numerous noises. However, from the comparison of the peak positions of the two electrodes, the Ni­(OH)_2_ electrode obviously has more extra peaks than those of the Ni­(OH)_2_+10%GWC electrode. Figure 4d shows the XRD patterns of the Ni­(OH)_2_ electrode without carbon addition before and after electrochemical testing to study the formation of NiOOH during the charge/discharge process. Except for the peaks from the nickel felt substrate, aluminum holder, and part of Ni­(OH)_2_, we can see some main peaks at 12.8, 25.9, 37.1, 37.9, 43.2, 58.7, 66.2, 68.2, and 79.1° corresponding to (0 0 3), (0 0 6), (1 0 1), (1 0 2), (1 0 5), (1 0 10), (1 1 0), (0 0 15), and (0 0 17) planes of hexagonal NiOOH (JCPDS card No. 06-0075). The NiOOH can be deduced to form during the charge/discharge process due to the redox reaction Ni^3+^/Ni^2+^. The peaks at 11.3, 33.4, 34.4, 38.7, 45.9, 60, and 61.2° correspond to (0 0 3), (1 0 1), (0 1 2), (0 1 5), (0 1 8), (1 1 0), and (1 1 3) planes of the α-Ni­(OH)_2_ powders (JCPDS card No. 38-0715). Besides, we also can observe the weak peaks of the Co­(OH)_2_ (JCPDS card No. 45-0031), CoO (JCPDS card No. 42-1300), and CoOOH (JCPDS card No. 42-1300) phases due to the small cobalt content (∼4%) in the Ni­(OH)_2_ and the oxidized process in the cell activation stage, and the weak redox reaction between Co^3+^/Co^2+^. Furthermore, the peak intensity of the Ni­(OH)_2_+10%GWC electrode is much lower than that of the Ni­(OH)_2_ electrode, which may be caused by the addition of GWC that can limit the formation of β-Ni­(OH)_2_ to α-Ni­(OH)_2_ and prevent the electrode expansion during the charge/discharge process. Due to the instability of α-Ni­(OH)_2_, it will also transfer back into the β-Ni­(OH)_2_ phase during the cycled process, which can be deduced according to the XRD results.

The state of charge (SOC) is one of the important parameters of the battery management system and is also the basis for evaluating the charge and discharge control strategy of a battery.^47^ Generally, the SOC cannot be obtained by direct measurement of the voltage due to the hysteresis and the complex structure of a battery.^48^ In this work, the open-circuit voltage (OCV) method is used for indirect testing and estimation. Figure 5a–c shows the open-circuit voltage hysteresis with a 100% SOC of different cells at 0.2C with different rest time after activation at 0.2C for 10 cycles. Also, Figure S7–9 (SI†) supports the results of different cells with different SOC depths. Generally, the conditions of a battery are relatively stable and then the functional relationship between the open-circuit voltage and the SOC is also relatively stable after it has been fully rested for a long waiting time. In this work, to save time, we compared different rest time during the charge/discharge process to test the open-circuit voltage. According to the results, when the rest time exceeds 4 min, the change in OCV is no more than 2%, thus it can be considered that the cell has reached a relatively stable state at this moment. As shown in Figure 5d, the open-circuit voltage hysteresis of different cells with 100% SOC at 0.2C with a rest time of 4 min were compared. Because when the rest time is 4 min, the change in OCV is around 0.5%, indicating a relatively stable state of the electrode. Although the difference in OCV during the charging process is small, it can still be seen that as the GWC content increases, the OCV of the corresponding cell is slightly lower, which indicates its better conductivity. At the same time, during the discharge process, when SOC is higher than 40%, they have the same rules as during charging. Electrodes with GWC show a lower voltage during discharge when SOC is below 40% which is unique among the tested carbon types. One might speculate that this is because the GWC particles are bigger than other carbon types and make fewer contact points with the nickel active material. The nickel active material is conductive due to the content of Ni^3+^. So, when extracting the electrons from fewer contact points one has to assume that the zone where the charge carriers are depleted becomes larger and more depleted.

**Figure 5:**
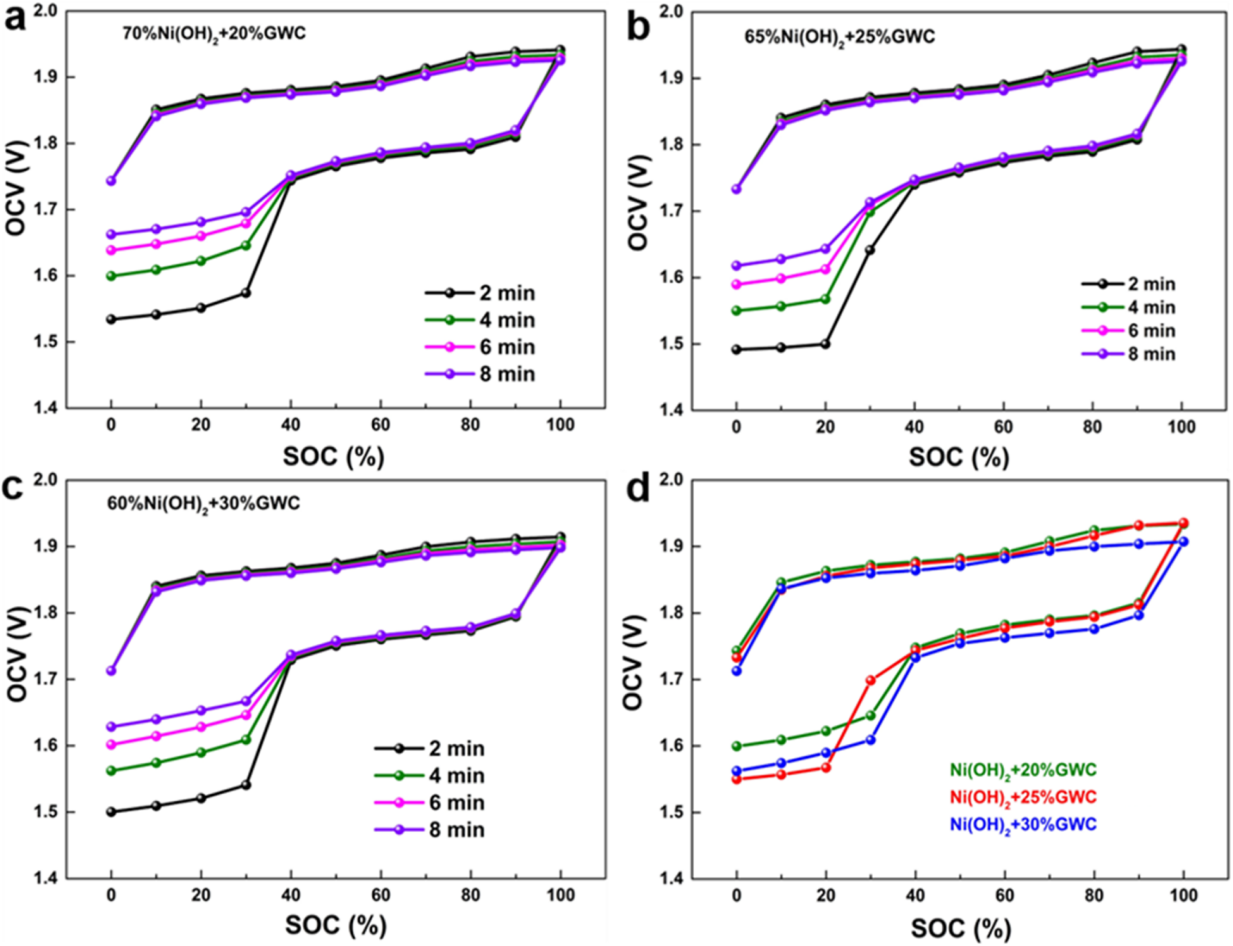
(a–c) The open-circuit voltage hysteresis and 100% state of charge of different cells with different Ni-electrodes containing different GWC contents at 0.2C and different rest time, and (d) the compared open-circuit voltage hysteresis of different cells at 0.2C at a rest time of 4 min.

To explore it clearly, we studied the SOC depths of different cells with different carbon additives (as shown in Figure 6). Compared with the sharply decreasing OCV of commercial carbon black around 1.7 V (Figure 6c), other cells containing carbons with different wood structures or graphite structures tend to have a platform. As shown in Figure 6b, the S, N codoped wood carbon has the same wood resource, but it has a higher specific surface area (140 m^2^ g^–1^, about 20 times than that of GWC) and lower conductivity after carbonization at lower temperature. Its loose and porous structure makes it easier to grind into finer particles and connect better with nickel hydroxide particles, resulting in good overall conductivity without a significant voltage drop of around 1.7 V. The other three cells with graphitic structure (Figure 6d–f) also show a stable voltage platform without obvious decreasing, demonstrating good conductivity. However, according to the SEM images of different carbon materials, the GWC has the largest size even after simply grinding. Therefore, the obvious voltage drop in the second platform of the SOC curve of the Ni­(OH)_2_+GWC//Zn cell may not be mainly due to the intercalation reaction of the ordered graphitic structure but is more likely to be generated by the poor overall electrode conductive network caused by the large broken wood carbon particle size. Notably, the unique voltage platform exhibited by the Ni­(OH)_2_+GWC//Zn cell in the low SOC region disappeared (Figure S10, SI†) after long cycling at different current rates (from 0.2 to 10C for 10 cycles, respectively), and the OCV vs SOC curve eventually became essentially consistent with other carbon additive cells. This phenomenon suggests a dual nature in the functional evolution of GWC. During low-current charge and discharge, the large-size GWC particles not only act as a conductive framework, but their unique woody hierarchical channels and partially graphitized structure may contribute additional interfacial capacitance or shallow ion storage at low currents, manifesting as a unique voltage platform. This characteristic aligns with some current reports suggesting that GWC can be used as an electrode material for energy storage. However, during long-term cycling, especially after high-current shocks, the mechanical stress generated by the repeated volume changes of the Ni­(OH)_2_ active material may disrupt the physical contact between it and the rigid GWC particles. This contact failure prevents the bulk capacitance contribution of the GWC particles from being effectively transferred to the external circuit, causing its unique voltage platform to disappear. Ultimately, the primary function of GWC in electrodes degenerates into providing a macroscopic conductive network, thus behaving similarly to other carbon materials that primarily rely on forming tight interfaces (such as carbon black and graphene). This observation also indirectly suggests that, in order to fully leverage the comprehensive advantages of GWC and maintain the stability of its long-term function, reducing its particle size and enhancing its mixing uniformity and contact strength with active materials through methods such as ball milling will be a key optimization direction in the future.

**Figure 6:**
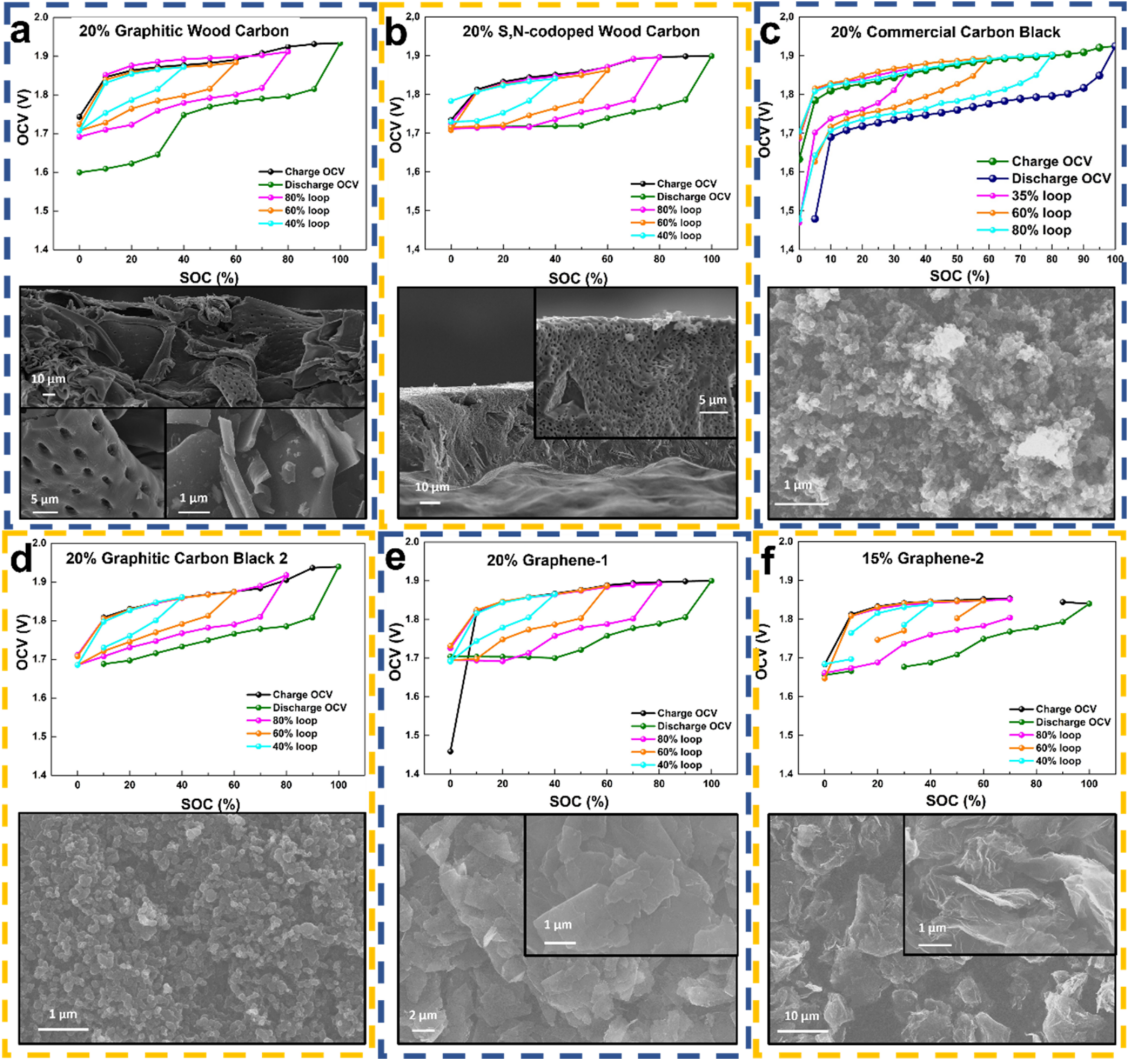
Open-circuit voltage hysteresis and state of charge of different cells with different carbon additives at 0.2C with a rest time of 4 min, and the corresponding SEM images of the carbons: GWC (a), S,N-codoped wood carbon (b), commercial carbon black (c), graphitic carbon black 2 (d), graphene-1 (e), and graphene-2 (f), respectively.

Furthermore, cyclic voltammetry (CV) measurements (Figure S11, SI†) were carried out to illustrate the performance of the fresh electrodes in a three-electrode system. As shown in Figure 7a, all the CV curves with different carbon additives at 0.2 mV s^–1^ present the typical couple of peaks corresponding to Ni^2+^/Ni^3+^ redox reaction. However, except for the pure Ni­(OH)_2_ electrode and the electrodes with carbon black 2 and graphitic carbon black 2 additives, the oxidation peaks of other electrodes are basically unobservable, and they all shift to the right beyond the forced potential window. This shows that in addition to CB2 and GCB2 purely enhancing conductivity, other carbon additives have additional capacitance contributions. In addition, after adding carbon materials, almost all reduction peaks shift to the left, indicating that it is more susceptible to reduction reactions, which is also a manifestation of enhanced conductivity. Moreover, the fully revealed reduction peaks are selected to compare the reaction kinetics of different electrodes. Generally, if there is a linear relationship between the peak current and the square root of the scan rate in the CV test, the slope of the straight line can quantitatively reflect the size of the diffusion coefficient.^49^ This process corresponds to the diffusion of electrolyte ions in the electrode material, and the larger the slope, the larger the electrolyte ion diffusion coefficient.^50,51^ As shown in Figure 7b, the electrodes containing graphitic carbon have larger slopes, indicating better reaction kinetics and higher diffusion coefficient of OH^–^ ions in the diffusion-controlled process. Also, according to this CV analysis, the addition of graphitized carbon improves the ion and electron transport within the cathode and improves its redox chemistry, enabling the battery to achieve better electrochemical performance.^52^

**Figure 7:**
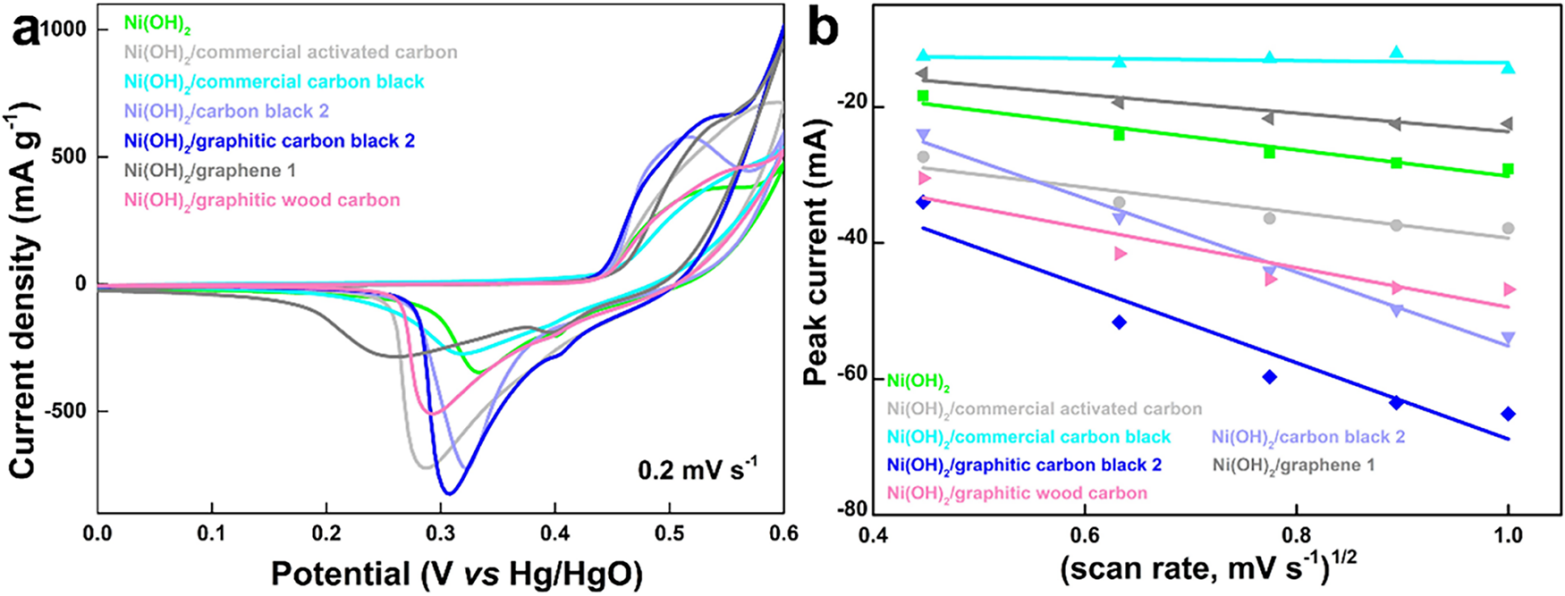
Electrochemical performance of different electrodes in a three-electrode system: (a) CV curves at 0.2 mV s^–1^, and (b) the relationship between the peak current and the square root of scan rate.

## 4 Conclusions

Different carbon qualities were evaluated as sustainable conductive additives to replace or reduce expensive cobalt and nickel additives in Ni­(OH)_2_ electrodes for aqueous rechargeable Ni–Zn batteries through a direct simple physical mixing process demonstrating the practicality of this approach. Graphitic wood-derived carbon showed the best performance among the evaluated carbon varieties. Optimization was conducted to find the best addition amount, and the optimal amount was about 25 wt %. We attributed this to a combination of the graphitic wood-derived carbon with its high conductivity, a partially graphitized carbon structure, and a large number of natural channels, and Ni­(OH)_2_ with a high theoretical capacity. The composite electrode exhibits good electrochemical performance, which can be attributed to a shorter ion transfer distance, faster ion diffusion rate, and lower electrochemical impedance. Moreover, the study of open-circuit voltage hysteresis and state of charge demonstrated that the graphitic wood-derived carbon can provide an additional ion intercalation/deintercalation to enhance the capacity during the charge/discharge process. This study indicates that graphitic wood-derived carbon is a promising low-cost and sustainable alternative to expensive metal powders in Ni­(OH)_2_ electrodes. The simple preparation process and the significant performance enhancement make GWC a highly attractive material for the development of more sustainable and cost-effective Ni–Zn batteries, and further provides a novel engineering solution for designing low-cost, long-life, cobalt-free nickel-based battery electrodes.

## Supplementary Material



## Notes

Additional digital photos, XRD patterns, and electrochemical performances for different cells are provided. Also, a table of the specific surface area and conductivity of some carbon additives (PDF)
