# Supplementary material for: Simple Physical Mixing of Graphitic Wood-Derived Carbon For High-Performance Ni(OH)2 Electrodes: A Sustainable Strategy Beyond Metal Additives
Source: ACS Omega. 2025 Dec 22;11(1):2170–80. doi: 10.1021/acsomega.5c11266 (PMC12809764; doi:10.1021/acsomega.5c11266)
Supplement: Supplementary file 1 [file ao5c11266_si_001.pdf]

Simple physical mixing of graphitic wood-derived carbon for high-performance $Ni(OH)_2$ electrodes: A sustainable strategy beyond metal additives

Xingyan Zhang*, Sadaf Saeedi Garakani, Gunder Karlsson, and Dag Noréus

Department of Chemistry, Stockholm University, SE 106 91 Stockholm, Sweden

*E-mail of the corresponding author: xingyan.zhang@su.se

Table S1 The specific surface area and conductivity of some carbon additives.

No.	BET (m^2/g)	Conductivity (S/m)
**Wood-derived carbon-1000**	**480**	**2000**
**Wood-derived carbon-2000**	**20**	**11000**
**S,N-doped wood-derived carbon**	**140**	**---**
**Commercial carbon black (CB),** Acetylene black (100% compressed)	**55-70**	**10000-25000**
**Commercial activated carbon (AC) TF-B520**	**1500-1800**	**2-10**

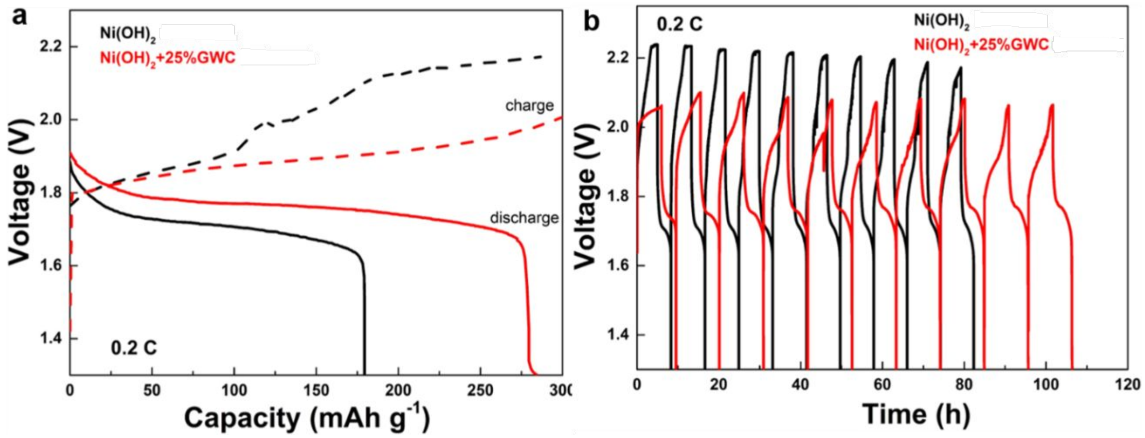

Fig. S1 Compared electrochemical performance of cells with/without 25%GWC.

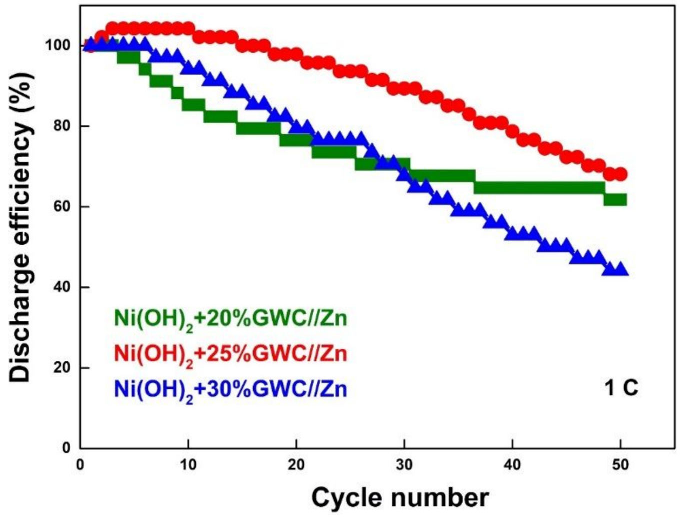

Fig. S2 Compared cycling performance of cells at 1 C.

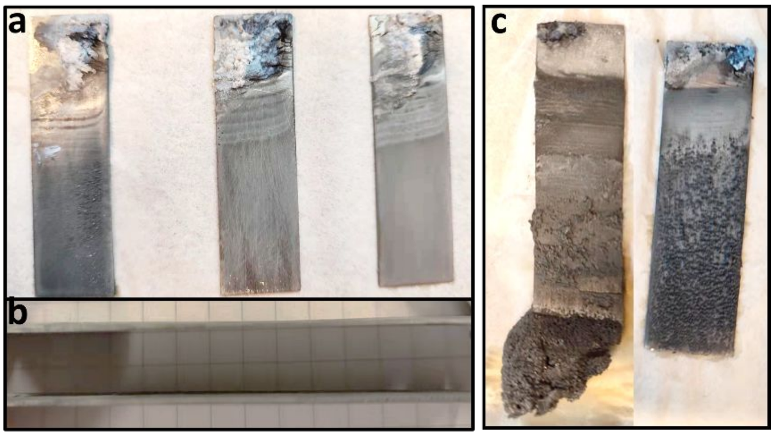

Fig. S3 Optical photos of zinc counter electrode: (a, b) surface and cross-section after one day of continuous testing, and (c) surface after two days of continuous testing at 1 C. The surface with more products corresponds to a lower carbon content in the Ni-electrode.

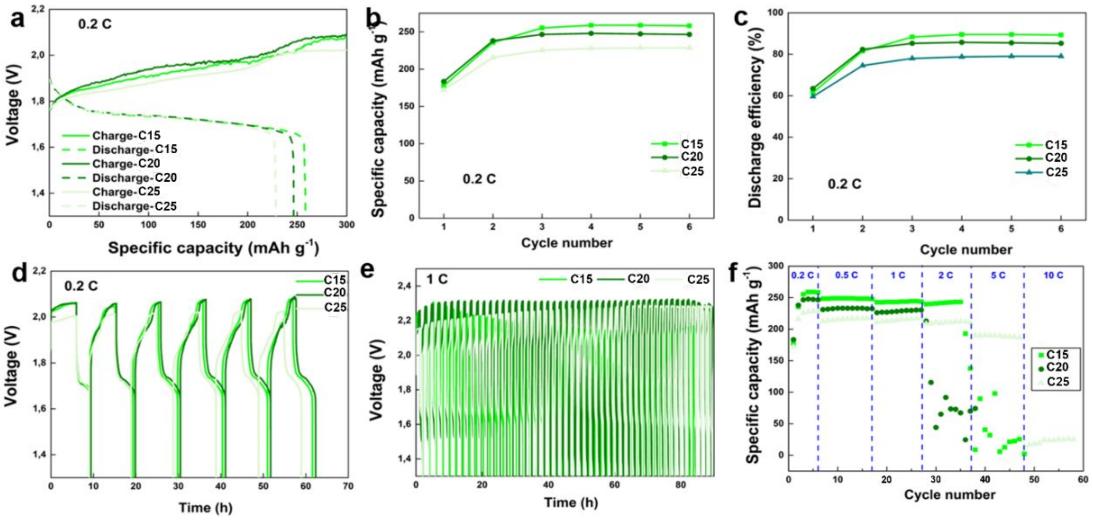

Fig. S4 Electrochemical performance of cells with different commercial carbon black contents (the apparent capacity fading at high rates is due to damage to the zinc anode during the charge/discharge process.)

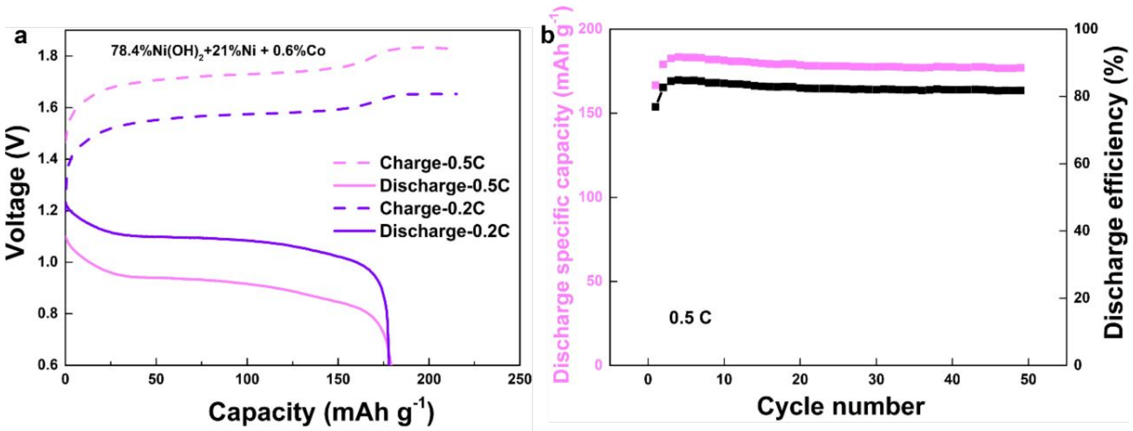

Fig. S5 Electrochemical performance of cells with Ni and Co metal powders.

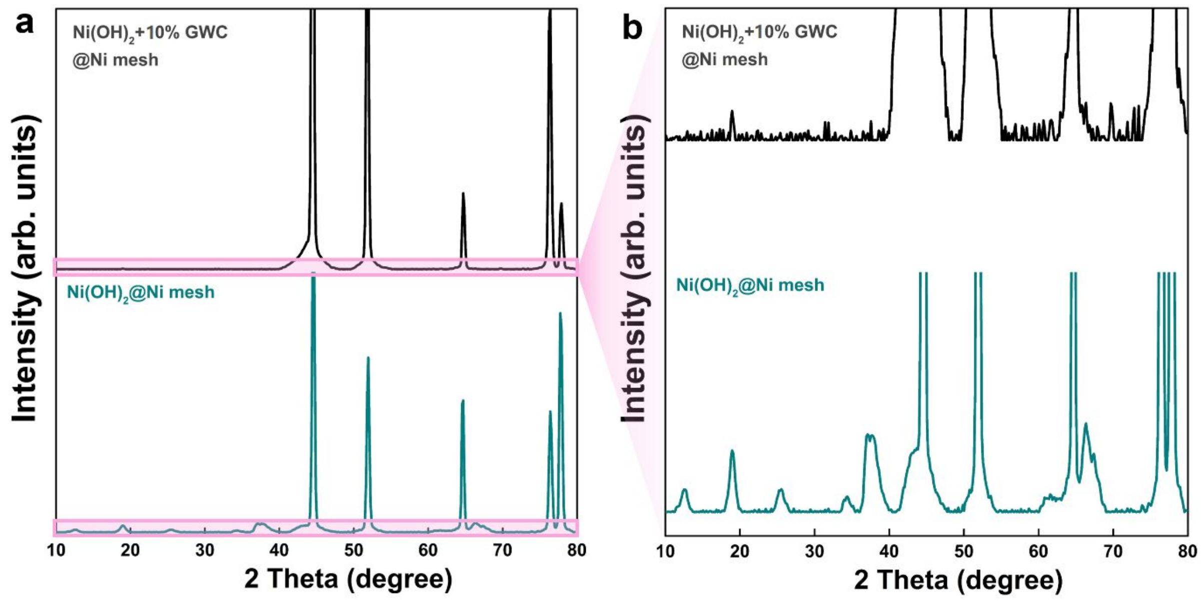

Fig. S6 XRD patterns of the $Ni(OH)_2$ and $Ni(OH)_2$+10% GWC electrodes after electrochemical testing.

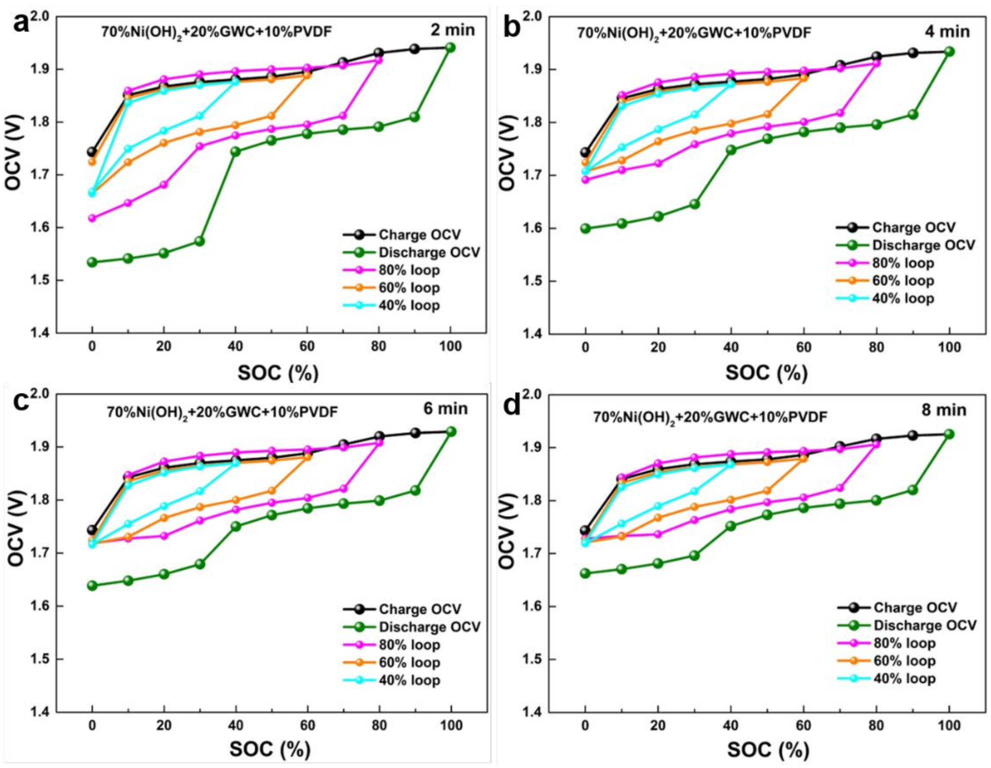

Fig. S7 The open-circuit voltage hysteresis and state of charge of the $Ni(OH)_2$+20%GWC//Zn cell at 0.2 C and different rested time: (a) 2 min, (b) 4 min, (c) 6 min, and (d) 8 min, respectively.

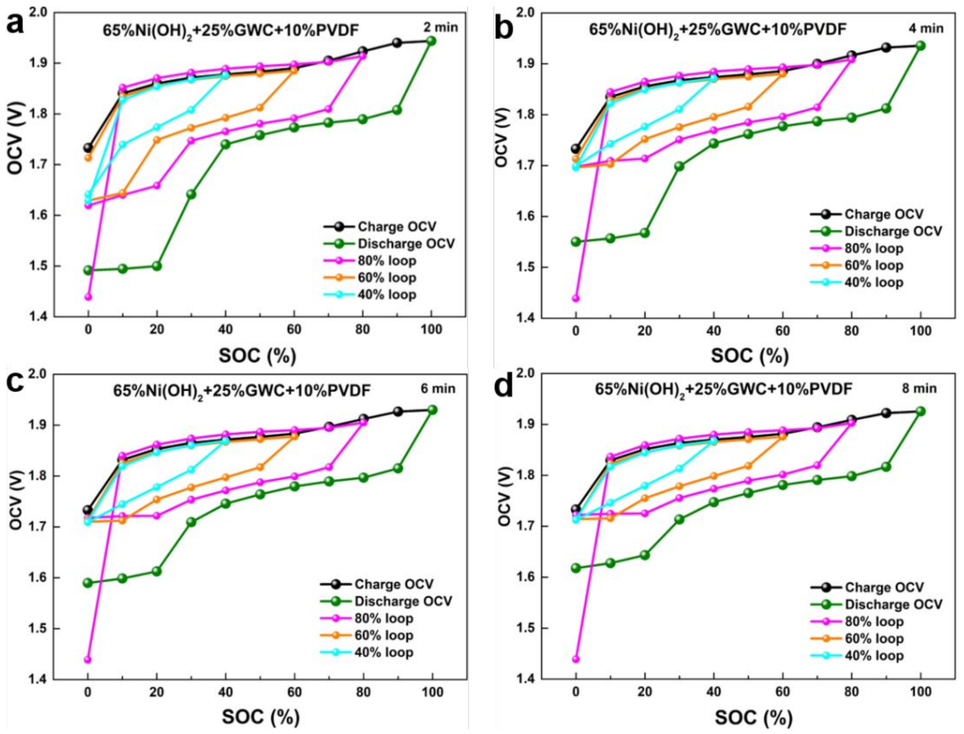

Fig. S8 The open-circuit voltage hysteresis and state of charge of the $Ni(OH)_2$+25%GWC//Zn cell at 0.2 C and different rested time: (a) 2 min, (b) 4 min, (c) 6 min, and (d) 8 min, respectively.

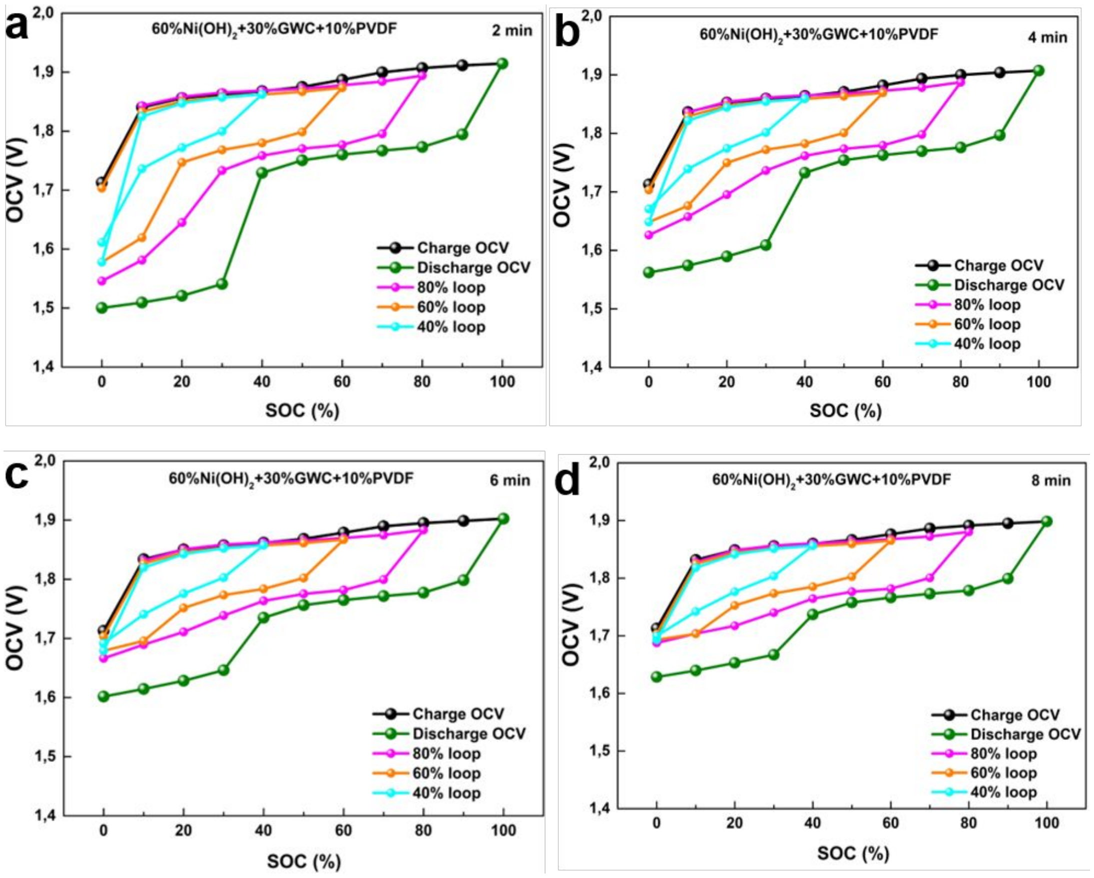

Fig. S9 The open-circuit voltage hysteresis and state of charge of the $Ni(OH)_2$+30%GWC//Zn cell at 0.2 C and different rested time: (a) 2 min, (b) 4 min, (c) 6 min, and (d) 8 min, respectively.

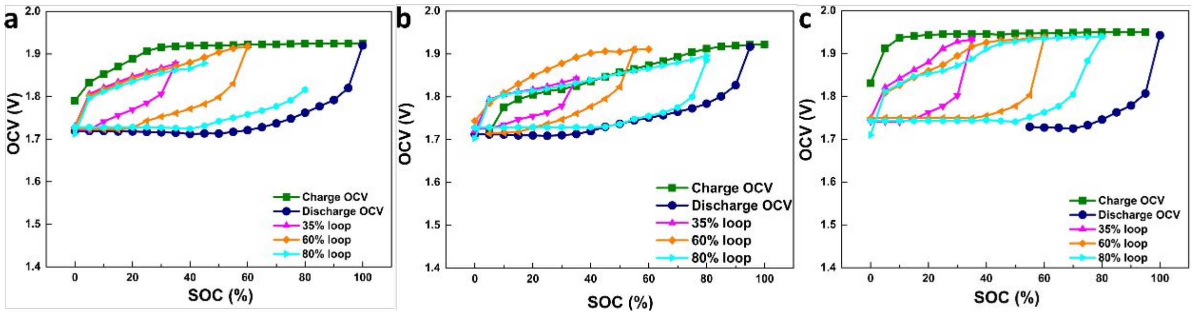

Fig. S10 The OCV of cells with different GWC (a: 20%, b: 25%, c: 30%) with different SOC window was tested after charge/discharge process at 0.2-10C for 10 cycles (total 60 cycles), respectively.

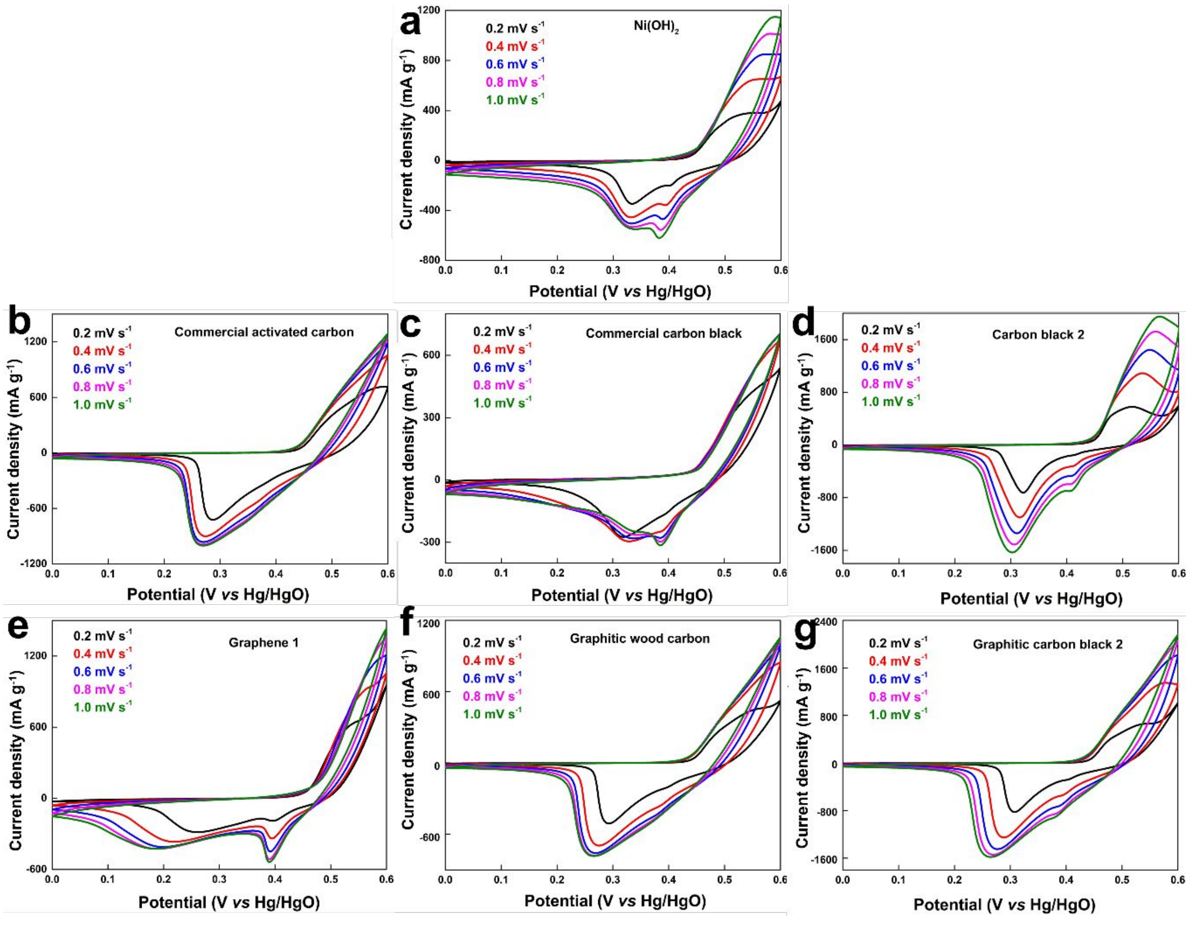

Fig. S11 CV curves of different electrodes based on $Ni(OH)_2$ with/without different carbon additives in a three-electrode system.
